# Massive Hydrothorax and Ascites as the Primary Manifestation of Infection With *Clostridium difficile*: A Case Report and Literature Review

**DOI:** 10.3389/fped.2020.00254

**Published:** 2020-05-20

**Authors:** Yujian Liang, Xiufang He, Ti Wang, Yili Chen, Huimin Huang, Wen Tang, Yijuan Li

**Affiliations:** ^1^Department of Pediatrics, The First Affiliated Hospital, Sun Yat-sen University, Guangzhou, China; ^2^Department of Pediatric Cardiology, Heart Center, The First Affiliated Hospital, Sun Yat-sen University, Guangzhou, China; ^3^Department of Laboratory Medicine, The First Affiliated Hospital, Sun Yat-sen University, Guangzhou, China

**Keywords:** *Clostridium difficile* infection, pediatric, large B-cell lymphoma, broad-spectrum antibiotics therapy, hydrothorax, ascites

## Abstract

**Introduction:** *Clostridium difficile* infection (cdi) often occurs with long-term and irregular use of antibiotics. Patients with tumors receiving both antibiotics and chemotherapy are at a high risk of cdi. The symptoms of cdi vary but can include diarrhea, hypovolemia, electrolyte imbalance, hypoproteinemia, toxic megacolon, gastrointestinal tract perforation, disseminated intravascular coagulation, sepsis, and other lethal complications. Here, we report a rare clinical manifestation associated with cdi in a child with lymphoma presenting with massive hydrothorax and ascites.

**Case Presentation:** A 6-year-old girl who was on chemotherapy for lymphoma presented with fever and was treated with intravenous broad-spectrum antibiotics 3 days before admission to our hospital. On the day before admission, she developed abdominal distension and diarrhea. After admission, broad-spectrum antibiotic therapy was initiated, and her hydrothorax and ascites were drained. An initial extensive microbiological evaluation revealed no pathogens, and laboratory tests and imaging studies of the pleural and peritoneal effusions revealed no evidence of cancer. The initial culture results for *C. difficile* were negative. The patient was diagnosed with CDI only after a positive test result for *C. difficile* toxin B gene and a repeated stool culture test revealed CDI. Intravenous antibiotics were suspended and replaced with oral vancomycin and *Saccharomyces boulardii*, which resulted in successful treatment and a good post-discharge outcome.

**Conclusions:** Massive hydrothorax and ascites are rare manifestations associated with CDI. CDI can occur in individuals with risk factors such as those undergoing broad-spectrum antibiotic therapy.

## Introduction

*Clostridium difficile* (CD) is an anaerobic gram-positive bacillus that is commonly detected in human and animal intestines and feces. Because of to the proliferation of toxigenic *C. difficile, C. difficile* infection (CDI) is a commonly reported cause of nosocomial intestinal infection, accounting for an estimated 20–30% of cases (1). Toxigenic *C. difficile* produces at least two exotoxins: toxin A, an enterotoxin, and toxin B, a cytotoxin. These two toxins attack the membrane or actin skeleton of intestinal mucosal cells, leading to diarrhea and enteritis. Long-term exposure to broad-spectrum antibacterial medication, especially clindamycin, quinolones, and third-generation cephalosporins; severe underlying diseases; low immunity; and inflammatory bowel disease (such as ulcerative colitis) are reported risk factors for CDI (2). Immunosuppressed cancer patients are at a high risk of CDI. CDI symptoms are variable and include diarrhea, hypovolemia, electrolyte imbalance, hypoproteinemia, toxic megacolon, gastrointestinal tract perforation, disseminated intravascular coagulation, sepsis, and other lethal complications; however, massive hydrothorax and ascites as the main manifestations of CDI are very rare. This report describes a child with a lymphoma who developed CDI and presented with massive hydrothorax and ascites. This is the first report of CDI as the cause of massive hydrothorax and ascites in a child with a tumor.

This case report was approved by the Ethics Review Board of the First Affiliated Hospital, Sun Yat-sen University. The patient and her parents provided written informed consent to publication of this report.

## Case Presentation

### Clinical Presentation

**Figure 1:**
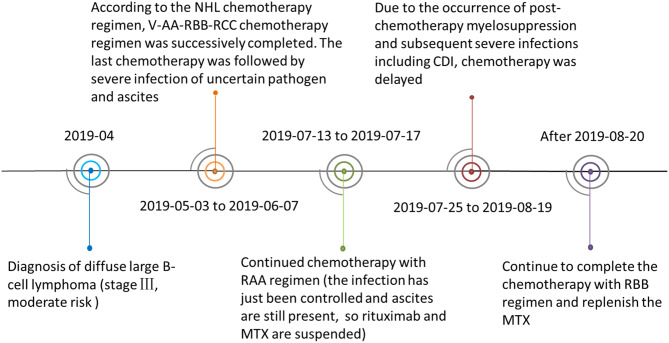
The patient's lymphoma chemotherapy timeline. Abbreviations: Ara-C, cytarabine; CDI, *Clostridiumdifficile* infection; CTX, cyclophosphamide; DXM, dexamethasone; IFO, ifosfamide; MTX, methotrexate; NHL, Non-Hodgkin's lymphoma; R, rituximab; VCR, vincristine; VP 16, etoposide. V-AA-RBB-RCC was the chemotherapy regimen combination used to treat the patient. V regimen: prednisone + CTX; AA regimen: DXM + IFO + VCR + Ara-C + MTX +VP 16; RBB regimen: R + DXM + CTX + VCR + MTX + Adriamycin; RCC regimen: R + DXM + vindesine + Ara-C +VP 16.

**Figure 2:**
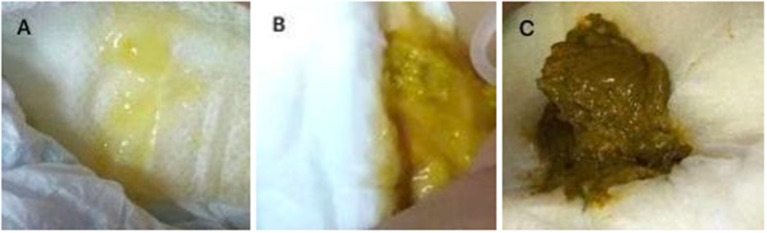
**(A)** Jelly-like stool from the patient before treatment; **(B)** Stool from the patient 3 days after the initiation of treatment; **(C)** Normal stool from the patient after 10 days of treatment.

A 6-year-old girl with lymphoma presented to our clinic with fever, diarrhea, abdominal distension, and shortness of breath, and was admitted. Before admission, she had received standard chemotherapy for a pediatric diffuse large B-cell lymphoma (3) at a specialist cancer hospital. The chemotherapy process is shown in Figure 1. Following the final chemotherapy session 10 days before admission, she had been in a period of post-chemotherapy myelosuppression, during which she developed a severe infection. After intravenous administration of cephalosporin antibiotics, she developed diarrhea and abdominal distension; however, no pathogen was identified. Her condition did not improve, and thus, the antibiotic therapy was discontinued. When the fever occurred during the period, she was administered intravenous vancomycin, imipenem, and carprofen for 3 days in a regional hospital, but her condition did not improve. Subsequently, she developed diarrhea with jelly-like stools (Figure 2), abdominal distension, nausea, vomiting, and shortness of breath. On Day 4, she was transferred to our clinic for further treatment.

### Clinical Findings

Clinical examination revealed the following findings on admission: temperature, 37.5°C; heart rate, 130 beats/min; respiratory rate, 52 breaths/min; respiratory moaning; neck and rib retraction; decreased bilateral breath sounds; shifting dullness (+); and mild edema of the lower limbs.

### Diagnostic Focus and Assessment

**Figure 3:**
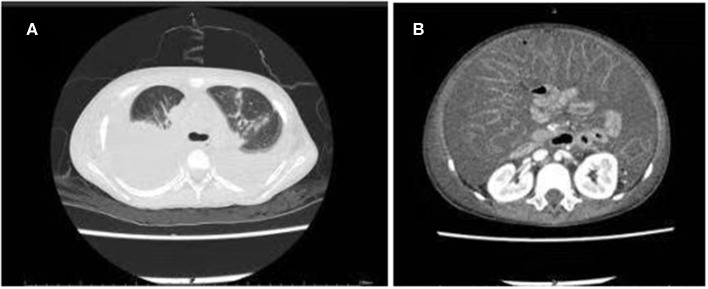
Computed tomography scan of the chest and abdomen showing multiple consolidations in both lung fields: **(A)** Pleural effusion and atelectasis bilaterally; **(B)** Massive ascites.

Laboratory tests performed on the day of admission revealed a white blood cell count of 1.80 × 10^9^/L (with 30.5% neutrophils); hemoglobin level of 6.9 g/dL; platelet count of 35 × 10^9^/L; elevated C-reactive protein levels of 220 mg/L; decreased albumin levels of 2.8 g/dL; and elevated lactate dehydrogenase levels of 282 U/L. A stool examination was positive for fecal white blood cells but was negative for common bacteria and fungiculture, rotavirus, adenoviridae antigens, and *C. difficile* culture using ChromID and *C. difficile* agar (CDIF) (bioMérieux, Marcy L'Étoile, France) (4). Chest and abdominal computed tomography showed lung consolidations, pleural effusion, bilateral atelectasis, and massive ascites (Figure 3).

Based on these findings, she was diagnosed with severe pneumonia and diarrheal disease; however, the cause of the massive hydrothorax and ascites remained unclear. She continued to be treated with vancomycin, imipenem, and carprofen, and she was mechanically ventilated for progressive dyspnea. To relieve her dyspnea, we performed bilateral drainage of the thoracic and abdominal effusions, and drained clear, yellowish fluid. The results of the laboratory tests performed on the pleural and peritoneal effusions are given in Table 1. With continued antibiotics and other therapy, her fever resolved. However, the amount of hydrothorax and ascites did not decrease significantly, and further tests were performed to investigate the cause of the persistent hydrothorax and ascites. A cytology test of the pleural and peritoneal fluid showed negative results. The chyle test, a qualitative test which uses ether to extract lipid and Sudan III to identify the presence of red fat globules microscopically, showed negative results, as did pathogen microbial high-throughput sequencing (PMseq). Bacteria, *Mycobacterium tuberculosis*, and fungal cultures of the pleural and peritoneal fluid were also negative. Due to the continuing diarrhea, we performed genetic testing using a real-time polymerase chain reaction assay for *C. difficile* toxin. This was positive for toxin B. To re-confirm the results, we re-cultured *C. difficile* using CDIF, and the test results were positive. Based on the above findings, we diagnosed CDI and hypothesized that the massive hydrothorax and ascites was a rare manifestation associated with the CDI.

**Table 1:** The fluid analysis of pleural and peritoneal effusion.

	**Hydrothorax**	**Ascites**
Rivalta test	Negative (-)	Negative (-)
White blood cell	260 × 10∧6/L	40 × 10∧6/L
Mononuclear	0.85%	0.90%
Polymorphonuclear	0.15%	0.10%
Total protein	30.2 g/L	29.8 g/L
Albumin	20.3 ge/L	21.4 g/L
LDH	309 U/L	335 U/L
Fluid/total protein	0.7	0.7
Fluid/LDH	0.9	1.1
Adenosine deaminase	4.6 U/L	6.1 U/L
High sensitive-CRP	105.73 mg/L	112.72 mg/L

*LDH, Lactate dehydrogenase; CRP, C-reactive protein*.

*The reference range of high sensitive-CRP is 0.10−3.0mg/L*.

### Therapeutic Focus and Assessment

CDI is an antibiotic-related infection; therefore, we discontinued intravenous antibiotic therapy following the diagnosis, and treated the patient with oral vancomycin and *Saccharomyces boulardii* immediately. Furthermore, her oncologist suggested that chemotherapy for her lymphoma be suspended until she recovered from the CDI.

### Follow-Up and Outcomes

Her stool frequency returned to normal after 3 days, her fever subsided after 1 week, her intestinal discomfort was relieved with the return to healthy stools, and her massive hydrothorax and ascites resolved completely. She was discharged after a 10-day oral course of vancomycin. She did not relapse during the 1-month follow-up period and resumed chemotherapy at the specialist cancer hospital.

## Discussion

CDI is commonly reported as a hospital-acquired infection. Patients with cancerous tumors who are receiving both antibiotics and chemotherapy have an increased risk of CDI. The incidence of CDI in hospitalized children in the United States is reported to have increased from 24.0/10,000 in 2003 to 58.0/10,000 in 2012, and the incidence in patients with cancer increased from 1.30/100 to 2.80/100 (11). According to data from a tertiary care hospital in China, the incidence of CDI in patients with hematologic tumors and bone marrow hematopoietic stem cell transplantation is 1.89/1000 and 3.69/1,000, respectively (9). It has previously been established that CDI is closely associated with imbalances in the intestinal flora, and patients with cancer are at risk of developing imbalances in their intestinal flora. Rajagopala et al. (5) investigated the intestinal flora of children with leukemia, and their results showed that the microbiota profiles of both the patient and control sibling groups were dominated by *Bacteroides, Prevotella*, and *Faecalibacterium*. At the genus level, both groups shared many taxa in common; however, the microbiota diversity in the patient group was significantly lower than that in the control group. Patients in an immunosuppressed post-chemotherapy state have also been reported to develop imbalanced intestinal flora (6). Chemotherapy used in the treatment of cancer can alter normal intestinal flora, causing extensive inflammatory changes in the intestinal tract and promoting both the growth of *C. difficile* and the production of its toxins (7). Our patient, with a lymphoma and history of chemotherapy and broad-spectrum antimicrobial medication use, had previously shown symptoms of intestinal flora disorders such as diarrhea and abdominal distension after her most recent course of chemotherapy, although we could not confirm whether she had an infection related to *C. difficile*. However, on her subsequent hospital admission, we confirmed that her diarrhea and abdominal distension were symptoms of CDI after testing for the *C. difficile* toxin gene. Moreover, we considered that her massive hydrothorax and ascites were associated with her CDI.

The clinical manifestations of CDI are diverse. Clinically, patients with mild infections may present with a single episode of diarrhea, which can be in the form of watery stools, green mucus stools, pus, and bloody stools. In severe cases, a patient is likely to defecate plaque-like false membranes, while jelly-like stools are rare. Severe infections often lead to fever, abdominal pain, bloating, diarrhea, and increased white blood cell counts accompanied by dehydration, hypoproteinemia, toxic megacolon, and sepsis. However, the presence of pleural and peritoneal effusions is a rare manifestation of CDI and may be misdiagnosed as cancerous effusions in patients with tumors. Most cases of CDI presenting with massive hydrothorax and ascites as the primary manifestation have been reported among immunodeficient adults, and reports in children are rare. A summary of the national and international studies (8, 10, 12–15) is listed in Table 2. Based on these studies, only eight patients had CDI that manifested as ascites, and only two patients had CDI manifesting as pleural effusions and ascites (one in a child, and one in an adult). The child developed CDI after the administration of amoxicillin for otitis media, and the clinical manifestations were chronic diarrhea (white mucus discharge), abdominal distension with ascites, massive right pleural effusion, and shock (12). The mechanism of massive hydrothorax and ascites due to CDI remains unclear; however, hypoalbuminemia, colonic inflammation with micro-perforation and infectious peritonitis, and toxin-mediated cytokines enhancing vascular permeability could be involved (8). Among the ten patients reported with CDI, nine had hypoproteinemia, which was reported to be severe in five patients. In our case, the child was immunosuppressed and had been exposed to broad-spectrum antibiotics, which are both risk factors for CDI. Therefore, we performed a *C. difficile* culture on admission using CDIF, which can isolate and identify *C. difficile* strains within 24 h with a high sensitivity (4). However, the *C. difficile* results were negative, which delayed the diagnosis. Given a high clinical suspicion for CDI, we re-cultured *C. difficile* using CDIF and performed genetic testing using a real-time polymerase chain reaction assay for *C. difficile* toxins, and both test results were found to be positive. The two main types of laboratory diagnosis for CDI comprise: (i) a fecal sample positive for *C. difficile* toxin and (ii) endoscopy or histopathological examination findings indicating pseudomembranous enteritis. *C. difficile* culture cannot be used to distinguish whether the strain has produced toxins; however, toxin detection is key to the diagnosis of CDI. At present, toxin detection methods include cell cytotoxicity assay, toxigenic culture, enzyme-linked immunoassay, and gene detection. Because gene detection has advantages in terms of rapidity, sensitivity, and specificity, genetic testing was used for our patient, despite the high cost of the test. Therefore, it is important to repeat tests, especially using different methods, when there is a high clinical suspicion of CDI.

**Table 2:** Summary of case studies with CD infection presenting with hydrothorax and ascites as main symptoms.

**Study by**	**Age**	**Primary disease**	**Antibiotics treatment before CDI/duration**	**Hydrothorax**	**Ascites**	**Antibiotic/duration**	**Outcome**
Pang et al. (5)	71 y	Gastrointestinal bleeding due to cirrhosis	Ceftriaxone IV/14 d	–	+	Ornidazole PO/14 d	cured
Shen et al. (6)	61 y	Ulcerative colitis/ 10d after intestinal operation	Ampicillin IV/NG	–	+	Vancomycin PO/NG Metronidazole IV/NG	died
Tsourous et al. (7)	60 y	Type 2 diabetes/ PAOD/soft tissue infection combined osteomyelitis	Amoxicillin, clindamycin PO/40 d	–	+	Vancomycin PO/NG	cured
Boaz et al. (8) Zukerman et al. (9)	25 y	Oral cavity infection	Clindamycin PO/10 d	+	+	Vancomycin PO/NG	cured
	54 y	AIDS/pneumocystosis	SMX, RMP PO/7 m	–	+	–	died
	48 y	Heroin IV usage/ URI	Erythromycin NG/7d	–	+	Metronidazole NG/NG	cured
	30 y	AIDS/ *P. aeruginosa* pneumonia	Ceftazidime, gentamicin IV/NG	–	+	Vancomycin PO/14d	cured
	33 y	AIDS	SMX, fluconazole NG /NG	–	+	Metronidazol/PO/14d	cured
	58 y	CAP	Erythromycin NG/6d	–	+	Metronidazole PO/21d	cured
Zwiener et al. (10)	2 y 6 m	Otitis media	Amoxicillin NG/10d	+	+	Vancomycin PO/14d	relapsed
Our case	6 y	Diffuse large B cell lymphoma	Vancomycin, imipenem IV/3d	+	+	Vancomycin PO/12d	cured

*CDI, Clostridium difficile infection; PAOD, peripheral arteriosclerotic occlusive disease; AIDS, acquired immune deficiency syndrome; URI, upper respiratory infection; CAP, community-acquired pneumonia; SMX, sulfamethoxazole; RMP, rifampicin; NG, not given; y, years; m, months; d, days; IV, intravenous*.

Early recognition of CDI is very important for its treatment, with timely suspension of the original antibiotics, application of antibiotics against *C. difficile*, administration of medication to adjust the intestinal flora, and rehydration and maintenance of electrolyte balance all being crucial in treating CDI. Diarrhea usually stops 5–8 days after discontinuing the original antibiotic; however, it has been reported to last for 2–3 weeks or even 2 months in some cases (16). When antibiotics are necessary to treat the primary disease, antibiotics that induce CDI, especially cephalosporins, clindamycin, and quinolones, should be avoided; narrow-spectrum antibiotics with minimal side-effects on the intestinal flora should be prescribed and administered parenterally, and the course of antibiotics should be as short as possible. If the antibiotics that induced CDI cannot be replaced or discontinued, a course of antibiotics against CDI should be extended to 1 week after the antibiotics that induced the CDI are stopped (17). Administration of metronidazole or vancomycin for 10 days is the preferred antibiotic therapy against *C. difficile*. For children with severe CDI, the use of oral vancomycin has been found to be associated with fewer relapses compared with metronidazole (18). In addition, probiotics can be used to adjust the gut microbiota. A systematic review of 38 randomized controlled trials (>8,000 patients) showed that Brassica yeast has anti-toxin effects in addition to adjusting the intestinal flora and has a better therapeutic effect on CDI than other probiotics (19). The use of intravenous immunoglobulins remains controversial (20). To treat hydrothorax and ascites due to CDI, it is necessary to treat the primary disease and to perform puncture and drainage when necessary, consistent with treatment for hydrothorax and ascites due to other causes. For our patient, after we obtained the diagnosis of CDI, all intravenous antibiotics were discontinued, and we switched her to oral vancomycin and Brassica yeast, which contributed to diarrhea improvement and the resolution of her massive hydrothorax and ascites. Furthermore, her chemotherapy for lymphoma was suspended as chemotherapy may aggravate CDI (7).

To conclude, massive hydrothorax and ascites are rare manifestations associated with CDI. Cancer patients who receive both chemotherapy and antibiotics are at a high risk of CDI. When clinical suspicion for CDI is high, repeat testing is important for early diagnosis and treatment.

## Data Availability Statement

All datasets generated for this study are included in the article/supplementary material.

## Ethics Statement

The studies involving human participants were reviewed and approved by The First Affiliated Hospital, Sun Yat-sen University. Written informed consent to participate in this study was provided by the participants' legal guardian/next of kin.

## Author Contributions

YL and XH are co-first author. YL conceptualized and designed the study, reviewed, and revised the manuscript. YL and XH carried out the initial analyses and drafted the initial manuscript. TW and HH were responsible for the treatment of the patient. YC and WT coordinated and supervised the data collection. All authors read and approved the final manuscript.

## Conflict of Interest

The authors declare that the research was conducted in the absence of any commercial or financial relationships that could be construed as a potential conflict of interest.
